# Pre-Dialysis Serum Myostatin Concentration Is Independently Associated with Intradialytic Muscle Cramps in Hemodialysis Patients

**DOI:** 10.3390/ijms27156929

**Published:** 2026-08-01

**Authors:** Agnieszka Makowka, Ewa Pawlowicz Szlarska, Gabriela Kot, Agata Wrobel, Michal Nowicki

**Affiliations:** 1 Department of Nephrology, Hypertension, Transplantation and Internal Medicine, Central University Hospital, Medical University of Lodz, Pomorska 251, 92-213 Lodz, Poland; 2 Faculty of Medicine, Medical University of Lodz, Pomorska 251, 92-213 Lodz, Poland

**Keywords:** hemodialysis, muscle cramps, myostatin, myokines

## Abstract

Muscle cramps are among the most common acute complications of hemodialysis (HD), often occurring even in the absence of electrolyte disturbances. Most cramps are painful and may cause patients to fear future dialysis sessions. We hypothesized that susceptibility to muscle cramps may be related to impaired skeletal muscle myokine secretion. No significant differences in pre-dialysis myokine levels were observed between patients with and without muscle cramps (myostatin 36.2 vs. 86.9 (*p* = 0.09), fibronectin type III domain-containing protein 5 (FNDC5) 0.6 vs. 0.6 (*p* = 0.61), interleukin-6 (IL-6) 2.4 vs. 2.0 (*p* = 0.4). However, after adjustment for lean tissue mass (LTM) and lean tissue index (LTI), myostatin levels were significantly lower in patients with cramps (0.80 [0.35–1.84] vs. 1.49 [0.45–6.31], respectively, (*p* = 0.048) for LTM, and 2.26 [1.04–4.06] vs. 3.89 [1.52–19.47], respectively, (*p* = 0.04) for LTI). In multivariable logistic regression analysis, log2-transformed myostatin concentration was independently and inversely associated with muscle cramps after adjustment for dialysis vintage, body mass index (BMI), and extracellular water. No association was found between muscle cramps and ultrafiltration volume or serum sodium and potassium levels. The study included 67 hemodialysis patients (HD patients), of whom 39 experienced repeated muscle cramps during dialysis. Muscle cramps were defined as painful involuntary muscle contractions lasting more than one minute during or shortly after a dialysis session. Plasma levels of myokines—myostatin, FNDC5, and IL-6—were assessed. Pre-dialysis myostatin, but not FNDC5 or IL-6, was independently associated with the occurrence of intradialytic muscle cramps. Further studies are needed to confirm its clinical utility.

## 1. Introduction

Hemodialysis causes rapid fluid and electrolyte shifts and is therefore linked to many acute complications, among which muscle cramps are particularly common and burdensome. They affect up to 33–86% of patients and can cause pain, anxiety before dialysis, and premature termination of sessions, thus significantly lowering quality of life [1,2,3,4,5,6,7,8].

Despite extensive research, the exact mechanism of intradialytic muscle cramps remains unclear but seems to be multifactorial. Proposed contributors include large ultrafiltration volumes, intradialytic hypotension with sympathetic activation, rapid pH changes leading to transient reductions in ionized calcium, and decreases in serum potassium and magnesium [1,3,7,8]. Other metabolic abnormalities such as carnitine, vitamin C deficiency, or elevated parathyroid hormone have also been implicated [3,6]. However, cramps frequently occur even in patients without major electrolyte disturbances or excessive ultrafiltration, suggesting additional unidentified mechanisms [2,7,9,10].

Preventive strategies, such as reducing ultrafiltration, modifying osmolality shifts, administering saline or mannitol, or supplementing electrolytes, vitamin E, or carnitine—have shown inconsistent effectiveness and some of them may compromise dialysis adequacy [1,3,6,7]. Notably, several studies have indicated that intradialytic exercise can reduce the frequency and severity of cramps, suggesting a potential role of impaired muscle function in their development.

Several studies have shown that intradialytic muscle cramps may occur less frequently and with lower intensity in patients who perform intradialytic physical exercise. This observation suggests that impaired skeletal muscle function may contribute to the pathogenesis of cramps [11,12].

As has been extensively studied during the last two decades, during physical activity, skeletal muscle cells release myokines to facilitate muscle fiber repair, modulate lipid and glucose metabolism, and inflammation. The most well-investigated studied myokines include myostatin, irisin, derived from the proteolytic cleavage of fibronectin type III domain-containing protein 5 (FNDC5) and IL-6. In hemodialysis patients, skeletal muscle function is often compromised due to metabolic acidosis, chronic inflammation, increased protein catabolism, and reduced physical activity. These factors promote sarcopenia and alter myokine secretion patterns [8,11,12,13,14,15,16,17,18,19,20].

Myostatin, a negative regulator of muscle mass, decreases during exercise but rises in conditions of immobilization or low muscle mass. Its levels increase with decreasing kidney function. Elevated myostatin may contribute to sarcopenia in hemodialysis patients, although its potential link to muscle cramps has not yet been investigated [11,12,14,15,16,18,20,21].

Irisin is a biologically active hormone cleaved from the FNDC5 protein and released into the bloodstream, predominantly during exercise or cold exposure, and is secreted by both muscle and adipose tissue. Its levels are markedly reduced in patients with chronic kidney disease and those undergoing hemodialysis. Low irisin concentrations have been associated with muscle atrophy, malnutrition, endothelial dysfunction, and vascular calcification; whereas, exercise-induced increases in irisin may improve muscle function, promote favorable adipose tissue remodeling, enhance bone metabolism, and reduce inflammation—mechanisms that could theoretically influence susceptibility to muscle cramps [11,16,17,20,22].

IL-6, a pro-inflammatory cytokine also classified as a myokine, is elevated in hemodialysis patients. Although IL-6 participates in muscle growth, repair, and metabolism, its specific role in intradialytic muscle cramps remains unclear [11,16,18,20].

Many studies have shown that introducing structured physical exercise during dialysis improved overall patient status, lowered cardiovascular risk, and reduced the frequency and severity of intradialytic muscle cramps. Building on this observation, we hypothesized that patients who report cramps may have impaired myokine responses, reflecting underlying skeletal muscle dysfunction. Such alterations in myokine secretion could contribute to cramp susceptibility in hemodialysis patients [11,15,23,24].

The aim of our study was to assess whether pre-dialysis serum levels of myostatin, FNDC5, and IL-6 are associated with the occurrence of intradialytic muscle cramps.

Identifying blood-based markers could help explain the link between muscle dysfunction and cramps, enable early identification of patients at risk, and support the development of targeted preventive strategies that improve patient comfort and dialysis adequacy.

## 2. Results

Sixty-seven HD patients were included. Thirty-nine people reported regular dialysis-related muscle cramps and 28 did not. Table 1 summarizes the clinical and biochemical characteristics of the patients.

All the patients were undergoing thrice-weekly HD. The most common causes of ESKD were glomerular disease (34%) and diabetic kidney disease (25%).

Incidence of muscle cramps did not significantly differ between males (60%) and females (55.6%); *p* = 0.72. There were no significant differences in median age, cause of end-stage kidney disease (*p* = 0.56), median dialysis vintage (*p* = 0.06) and mean time of dialysis session (*p* = 0.62) between patients experiencing muscle cramps and those who did not report this symptom.

No significant differences were observed with regard to interdialytic body mass gain and incidence of intradialytic hypotension episodes between patients with and without muscle cramps. Mean systolic and diastolic blood pressure values before the dialysis session also did not differ between the groups, similarly no differences were found with regard to potassium and calcium levels before the dialysis session.

Body composition parameters in patients with and without muscle cramps are presented in Table 2.

BMI was significantly higher in patients experiencing muscle cramps than in those who did not report cramps (26.6 vs. 23.1 *p* = 0.002). In addition to higher BMI, patients experiencing muscle cramps during hemodialysis also had higher TBW and ECW compared to patients without cramps (44.9 vs. 38.7 *p* = 0.041 and 20.7 vs. 17.1 *p* = 0.017).

No significant relation was revealed between the presence of muscle cramps and the level of hydration or serum sodium and potassium concentration.

There was no significant difference between the pre-dialysis concentration of myokines in patients reporting muscle cramps and those without this complication (Table 3).

**Figure 1:**
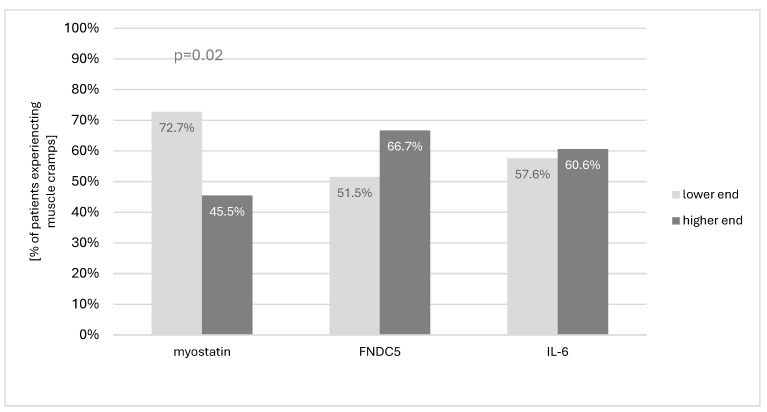
Comparison of incidence of muscle cramps among groups according to median of serum myostatin, FNDC5 and IL-6.

Since no significant difference in myokine concentrations was observed between patients reporting muscle cramps and those without this complication, and because the absolute values displayed considerable variability, patients were subsequently divided into two subgroups based on the median myostatin concentration (48 pg/mL). In this analysis, the lower-myostatin subgroup demonstrated a significantly higher incidence of muscle cramps. No such difference was seen in case of FNDC5 and IL-6 plasma concentration (Figure 1).

In addition, adjustment of all myokines to lean tissue mass (LTM) and lean tissue index (LTI), which reflect the amount of non-fat tissues in the body and its standardization for a person’s height, respectively, was performed. These indicators turned out to be significantly different according to presence of muscle cramps for myostatin, but not for FNDC5 and IL-6 (Table 4).

In logistic regression analysis, log-transformed myostatin concentration showed borderline inverse association with muscle cramps in unadjusted model; however, it became significant after adjustment for dialysis vintage, BMI, and ECW, which differed significantly between patients with and without muscle cramps (Table 5).

**Figure 2:**
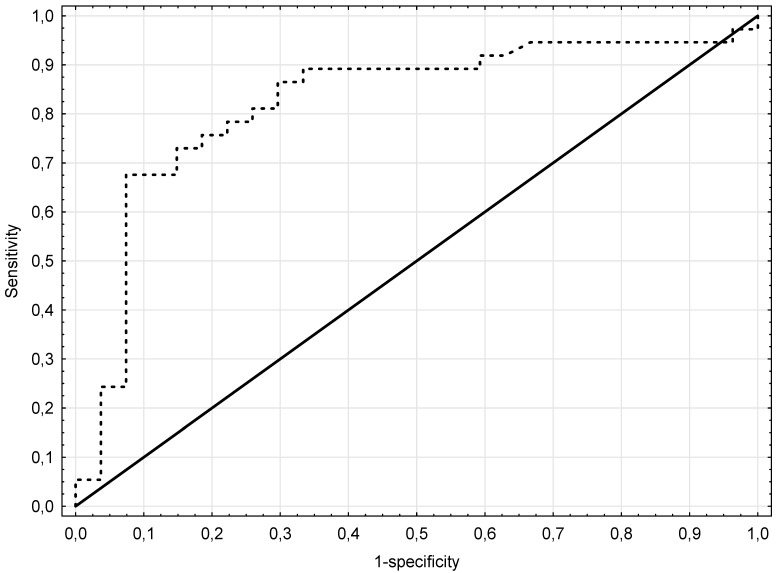
ROC curve for logistic regression model (log-transformed myostatin, adjusted for dialysis vintage, BMI and ECW).

ROC curve for model 3 showed sensitivity of 86.5%, specificity of 70.4%, with AUC of 0.823 (AICc value—79.2) (Figure 2).

## 3. Discussion

Although several studies have suggested potential links between rapid fluid shifts, high ultrafiltration rates, intradialytic hypotension, and electrolyte disturbances and the occurrence of painful muscle cramps during hemodialysis [1,2,3,6,7,8,9,10], our analysis initially did not confirm any significant association between these factors and cramps risk. However, after adjustment for serum myostatin concentration, a significant association emerged, indicating that the relationship may be influenced by muscle-related factors.

In this study, we aimed to evaluate whether circulating myokines are associated with the occurrence of painful intradialytic muscle cramps. Given the high prevalence of sarcopenia in this population, altered myokine secretion could represent a biologically plausible mechanism linking muscle metabolism with the occurrence of cramps. Although myostatin is traditionally regarded as a negative regulator of muscle growth, circulating concentrations may also reflect overall muscle metabolic activity and skeletal muscle health. Lower serum myostatin levels in dialysis patients have previously been associated with reduced muscle mass, protein-energy wasting, and sarcopenia. Consequently, lower myostatin concentrations may represent a marker of impaired muscle integrity and increased susceptibility to neuromuscular dysfunction, thereby contributing to the occurrence of intradialytic muscle cramps. Our results suggest that myostatin, but not FNDC5 and IL-6, may be considered as a potentially relevant biomarker associated with muscle cramps [11,13,18,21].

To the best of our knowledge, this is the first study assessing these relationships in hemodialysis patients. Interestingly, we observed an inverse association between BMI and the occurrence of muscle cramps, suggesting that patients with higher BMI may be less prone to cramp episodes. Although direct evidence linking BMI to intradialytic muscle cramps is lacking, this observation may reflect the so-called “obesity paradox” described in hemodialysis populations, in which higher BMI has been associated with improved survival and clinical outcomes. This effect has been attributed to greater metabolic and nutritional reserves, as well as a potential protective role of preserved muscle mass [25,26].

We did not find a difference between unadjusted myostatin concentration in patients with and without cramps, however after its correction for lean tissue mass and dialysis vintage, BMI and body water distribution (ECW) serum myostatin was independently and inversely associated with intradialytic muscle cramps.

A possible explanation for this finding could be that uncorrected serum myostatin levels may not reflect its biological activity in the hemodialysis patient population, as their status is influenced by changes in body composition, namely muscle mass, which is the source of body production of this myokine, and fluid status. In addition, biomarker levels were measured before dialysis; whereas, muscle cramps are acute events that typically occur during or toward the end of the session. This temporal mismatch may have limited our ability to capture some dynamic changes preceding cramps onset. Furthermore, muscle cramps are transient and multifactorial phenomena; whereas, circulating myokine levels are more likely to reflect chronic alterations in muscle mass and systemic inflammation rather than acute neuromuscular excitability.

Myokines such as myostatin, irisin, and IL-6 are influenced by multiple confounders, including physical activity, nutritional status, inflammation, and comorbidities. It is possible that myostatin, as a negative regulator of muscle growth contributing to sarcopenia, may influence susceptibility to cramps but does not directly trigger their occurrence. In this context, we explored the relationship between circulating myokines and fat-free mass as a surrogate measure of muscle quantity, which showed significant differences.

Previous studies in hemodialysis populations have demonstrated a positive association between physical activity and irisin-related pathways. Irisin is a peptide derived from proteolytic cleavage of the membrane protein FNDC5. However, its direct measurement is still controversial methodologically, as commonly used tests, especially ELISA, may not be specific and reliable enough in humans [27]. For this reason, we assessed FNDC5 rather than circulating irisin, as it may represent a more stable marker of pathway activation. Our findings are consistent with reports showing that acute exercise does not necessarily induce measurable short-term changes in this axis, suggesting a limited role in acute neuromuscular excitability underlying cramp development [17,22].

Noteworthy, myokine measurements limited to pre-dialysis sampling may not adequately capture intradialytic fluctuations. Available evidence suggests that a single hemodialysis session does not markedly affect circulating myostatin levels, supporting the relative stability of this biomarker. Additionally, the subjective nature of cramp reporting, without objective monitoring, may introduce variability and reduce the sensitivity of the analysis [13,18].

Individual variability in muscle reactivity, electrolyte balance, inflammatory status, body composition, and physical activity may further contribute to our findings.

Overall, lower serum myostatin concentrations were independently associated with intradialytic muscle cramps after adjustment for body composition parameters; whereas, FNDC5 and IL-6 were not significantly associated with muscle cramp occurrence in our study.

Due to the cross-sectional design of the present study, causal relationships cannot be established and the predictive performance of serum myostatin cannot be determined. Although lower myostatin concentrations were independently associated with intradialytic muscle cramps, prospective longitudinal studies are required to evaluate whether serum myostatin has true predictive value and potential clinical utility.

Another limitation of this study is that circulating myokine concentrations were measured only once before the dialysis session. Since intradialytic muscle cramps typically occur during or towards the end of treatment, dynamic changes in myokine levels during dialysis were not assessed. Therefore, the potential relationship between intradialytic fluctuations in myokines and cramp occurrence remains unknown and warrants further investigation.

Prospective studies incorporating serial intradialytic biomarker measurements are needed to further elucidate the relationship between myokines and intradialytic muscle cramps.

Muscle cramps were identified based on patient-reported questionnaire responses and were not objectively confirmed by clinical observation or electrophysiological assessment. Therefore, recall bias and subjective symptom reporting cannot be excluded.

Finally, our research is limited by the relatively small sample size, potential heterogeneity of the study population, and the lack of intradialytic biomarker measurements. Future studies should focus on dynamic assessment of myokines during dialysis sessions and incorporate objective monitoring of muscle cramps.

Our findings highlight the complexity of muscle cramps pathophysiology and suggest that serum biomarkers alone are insufficient to explain this phenomenon in hemodialysis patients.

## 4. Materials and Methods

This study was carried out in accordance with the ethical standards set forth in the Declaration of Helsinki and its later amendments. The research protocol received approval from the institutional ethics committee, and all participants provided written informed consent prior to enrollment.

The study was cross-sectional and was performed in a single tertiary nephrology center. The study group included 67 adult patients with end-stage kidney disease (ESKD) treated by chronic hemodialysis for at least 6 months (40 men, 27 women, mean age 66 ± 25 years).

The exclusion criteria included a positive history of kidney or any other solid organ transplantation, acute or chronic infection, decompensated non-respiratory acidosis, cancer, symptomatic heart failure with reduced ejection fraction, liver disease, bone-mineral disorder other that those associated with chronic kidney disease, primary muscular disease, uncontrolled arterial hypertension, significantly limited physical activity due to disease of the nervous, muscular or skeletal system.

After signing an informed consent, each patient was asked to complete a questionnaire containing nine closed questions addressing the occurrence of intra- and interdialytic muscle cramps. These questions concerned the duration of dialysis therapy, changes in body mass during the last dialysis sessions and the occurrence of muscle cramps during dialysis and in the interdialytic period, their duration and intensity (Table 6).

An episode of muscle cramps was defined as the painful involuntary muscle contraction that lasted for more than one minute during hemodialysis [2].

Before the mid-week dialysis session, body composition was measured using multifrequency bioimpedance spectroscopy with the Body Composition Monitor (BCM; Fresenius, Bad Homburg, Germany). Blood samples were collected immediately before and after this dialysis session to assess serum concentrations of calcium, potassium, magnesium, myostatin, irisin, and interleukin-6.

Body mass was measured before and after hemodialysis. In addition, blood pressure was measured before, in the middle and at the end of the session. Blood pressure was also additionally measured in the event of deterioration of well-being in order to assess an episode of intradialytic hypotension (IDH), which was classified according to the current definition as a significant drop in systolic blood pressure by ≥20 mmHg or mean blood pressure by ≥10 mmHg, occurring during or shortly after a dialysis session, which requires intervention.

### Statistical Analysis

The results are presented as mean ± standard deviation (SD) or median and interquartile range (IQR) for normally and non-normally distributed variables, respectively. The distribution of continuous variables was assessed with Shapiro–Wilk test. Depending on the variable distribution, t-test or Mann–Whitney U test was used for comparisons between two independent groups. Rank Spearman test was applied to assess non-parametric correlations. Pearson chi-square and Fisher exact tests were used to assess associations between categorical variables. To independently evaluate the factors associated with muscle cramps incidence, univariate and multivariate logistic regression analyses were performed. After univariate screening, variables with significant associations were entered into a multivariable logistic regression model with muscle spasms as the outcome. Correlations between chosen continuous variables were tested to avoid collinearity. Additionally, a receiver operating characteristic (ROC) curve was generated to assess the model’s discriminative ability, area under the ROC curve, sensitivity and specificity were determined. Corrected Akaike Information Criterion (AICc) was used to evaluate model quality. An α-level of *p* < 0.05 was set as statistically significant. Statistical analysis was performed and graphs were plotted using Statistica version 13.1 PL software (TIBCO Software Inc, Palo Alto, CA, USA).

## Figures and Tables

**Table 1:** Characteristics of the entire study group and subgroups with and without intradialytic cramps.

Variable	Total (n = 67)	With Cramps (n = 39)	Without Cramps (n = 28)
Age, years	66 (46–71)	69 (48–73)	64 (44–69)
Sex (men), n (%)	40 (59.7%)	24 (61.5%)	16 (57.1%)
Dialysis vintage, months	22.6 (12–57)	20.4 (12–38.8)	42 (13.1–59.7)
Cause of ESKD, n (%)			
DKD, n (%)	17 (25.4%)	12 (30.1%)	5 (17.9%)
Primary glomerular disease, n (%)	23 (34.2%)	14 (35.9%)	9 (32.1%)
Hypertensive kidney disease, n (%)	5 (7.5%)	3 (7.7%)	2 (7.1%)
Renal vascular disease n (%)	1 (1.5%)	0 (0%)	1 (3.6%)
ADPKD, n (%)	5 (7.5%)	3 (7.7%)	2 (7.1%)
Other/unknown, n (%)	16 (25.4%)	7 (17.9%)	9 (32.1%)
Duration of each dialysis session, minutes	216 ± 29.7	217 ± 31.7	213 ± 27.1
Systolic blood pressure, mmHg	141.8 ± 24.3	141.7 ± 23.2	142.1 ± 26.2
Diastolic blood pressure, mmHg	78.3 ±16.7	76.5 ± 17.2	80.8 ± 15.9
Intradialytic hypotension, n (%)	18 (26.9%)	10 (25.6%)	8 (28.6%)
BMI, kg/m^2^	25.1 (22.4–28.6)	26.6 (24.1–30.3)	23.1 (20.9–25.7)
Interdialytic body mass gain, kg	1.8 (1.1–2.8)	1.8 (1.1–3.1)	1.8 (1.2–2.7)
Overhydration, L	1.3 (0.2–2.9)	2.5 (0.2–3.0)	0.7 (0.2–2.7)
Urea distribution volume, L	41.1 (34.2–48.9)	42.7 (35.7–51.0)	38.3 (33.3–42.7)
TBW, L	41.5 (36.7–50.5)	44.9 (38.1–52.0)	38.7 (34.4–43.1)
ECW, L	18.4 (16.5–23.3)	20.7 (17.7–24.6)	17.1 (15.2–19.8)
ICW, L	23.1 (19.1–27.9)	24.2 (19.6–28.1)	21.9 (18.6–27.4)
E/I,	0.8 ± 0.1	0.9 ± 0.1	0.8 ± 0.1
LTM, kg	49.7 ± 12.6	51.7 ± 12.8	46.7 ± 12
LTI, kg/m^2^	17.1 (14.4–19.9)	17.6 (14.4–20.4)	15.9 (14.6–19.0)
Fat mass, kg	15.9 (12.1–21.3)	18.2 (13.5–24.2)	14.2 (11.8–19.1)
FTI, kg/m^2^	8.2 (6.3–10.9)	9.1 (6.2–11.2)	7.6 (3.6–9.2)
ATM, kg	22.8 (17.6–29)	24.9 (18.3–32.9)	19.4 (16.2–26.0)
BCM, kg	28.9 ± 8.7	30.5 ± 8.9	26.5 ± 8
Serum potassium, mmol/L	5.1 ± 0.6	5 ± 0.5	5.2 ± 0.7
Serum total calcium, mmol/L	2.2 ± 0.2	2.2 ± 0.2	2.2 ± 0.2
Serum myostatin, pg/mL	48 (23.9–124.5)	36.2 (19.1–107.5)	87 (25.4–178)
Serum FNDC5, ng/mL	0.6 (0.5–0.7)	0.6 (0.5–0.7)	0.6 (0.5–0.7)
Serum IL-6, pg/mL	2.2 (1.7–3)	2.4 (1.8–3)	2 (1.5–3)

Discrete data are presented as counts (n) and percentage of the total (%), while continuous data are presented as mean ± SD or median (Q1–Q3). Abbreviations: ESKD—end-stage kidney disease; DKD—diabetic kidney disease; ADPKD—autosomal dominant polycystic kidney disease; BMI—body mass index; TBW—total body water; ECW—extracellular water; ICW—intracellular water; E/I = extracellular water/intracellular water ratio, LTM—lean tissue mass; LTI—lean tissue index; FTI—fat tissue index; ATM—adipose tissue mass; BCM—body cell mass; FNDC5—fibronectin type III domain-containing protein 5; IL-6—interleukin 6.

**Table 2:** Body composition parameters in patients with and without muscle cramps.

Variable	Muscle Cramps (n = 39)	No Muscle Cramps (n = 28)	*p* Value
BMI, kg/m^2^	26.6 (24.1–30.3)	23.1 (20.9–25.7)	**0.002**
Overhydration, L	2.5 (0.2–3.0)	0.7 (0.2–2.7)	0.252
Urea distribution volume, L	42.7 (35.7–51.0)	38.3 (33.3– 42.7)	0.059
TBW, L	44.9 (38.1–52.0)	38.7 (34.4–43.1)	**0.041**
ECW, L	20.7 (17.7–24.6)	17.1 (15.2–19.8)	**0.017**
ICW, L	24.2 (19.6–28.1)	21.9 (18.6–27.4)	0.312
E/I,	0.9 ± 0.1	0.8 ± 0.1	0.177
LTM, kg	51.7 ± 12.8	46.7 ± 12	0.124
LTI, kg/m^2^	17.6 (14.4–20.4)	15.9 (14.6–19.0)	0.227
Fat mass, kg	18.2 (13.5–24.2)	14.2 (11.8–19.1)	0.080
FTI, kg/m^2^	9.1 (6.2–11.2)	7.6 (3.6–9.2)	0.258
ATM, kg	24.9 (18.3–32.9)	19.4 (16.2–26.0)	0.098
BCM, kg	30.5 ± 8.9	26.5 ± 8	0.078

Continuous data are presented as mean ± SD or median (interquartile range). Abbreviations: BMI—body mass index; TBW—total body water; ECW—extracellular water; ICW—intracellular water; E/I = extracellular water/intracellular water ratio, LTM—lean tissue mass; LTI—lean tissue index; FTI—fat tissue index; ATM—adipose tissue mass; BCM—body cell mass.

**Table 3:** Comparison of serum myokine levels in hemodialysis patients with and without muscle cramps.

Myokine	Muscle Cramps (n = 39)	No Muscle Cramps (n = 28)	*p* Value
Myostatin	36.2 (19.1–107.5)	86.9 (25.4–178)	0.09
FNDC5	0.6 (0.5–0.7)	0.6 (0.5–0.7)	0.61
IL-6	2.4 (1.8–3)	2 (1.5–2.9)	0.40

Continuous data are presented as median (interquartile range). Abbreviations: fibronectin type III domain-containing protein 5 (FNDC5) and interleukin-6 (IL-6).

**Table 4:** Comparison of serum myokine levels adjusted to LTM and LTI in hemodialysis patients with and without muscle cramps.

Adjusted Serum Myokines	Muscle Cramps (n = 38)	No Muscle Cramps (n = 25)	*p* Value
Myostatin/LTM	0.80 (0.35–1.84)	1.49 (0.45–6.31)	**0.048**
Myostatin/LTI	2.26 (1.04–4.06)	3.89 (1.52–19.47)	**0.040**
FNDC5/LTM	0.01 (0.01–0.02)	0.01 (0.01–0.02)	0.628
FNDC5/LTI	0.04 (0.03–0.04)	0.04 (0.03–0.05)	0.828
IL-6/LTM	0.05 (0.04–0.07)	0.05 (0.03–0.07)	0.720
IL-6/LTI	0.13 (0.10–0.19)	0.13 (0.10–0.18)	0.763

Continuous data are presented as median (Q1–Q3). Abbreviations: fibronectin type III domain-containing protein 5 (FNDC5) and interleukin-6 (IL-6), LTM—lean tissue mass, LTI—lean tissue index, Q1—first quartile, Q3—third quartile.

**Table 5:** Logistic regression analyses for the associations of log-transformed myostatin with muscle cramps.

	Myostatin		
	OR	95% CI	*p* Value
Muscle cramps			
Crude	0.745	0.544–1.022	0.068
Model 1	0.665	0.473–0.935	0.019
Model 2	0.689	0.478–0.993	0.046
Model 3	0.644	0.437–0.950	0.026
Crude	Log-transformed variable.		
Model 1	Crude + dialysis vintage		
Model 2	Model 1 + BMI		
Model 3	Model 2 + ECW.		

Abbreviations: BMI—body mass index; ECW—extracellular water.

**Table 6:** A questionnaire designed to assess the incidence of muscle cramps in chronic hemodialysis patients.

**1. How long have you been on dialysis:**
1. 0-3 months
2. 3-6 months
3. 6-12 months
4. Over 1 year
2. Has your dry weight been changed in the last month?
1. Yes
2. No
3. I don’t know
3. Has your dialysis time been changed in the last month?
1. Yes
2. No
3. I don’t know
4. Do you experience muscle cramps during dialysis session?
1. Yes
2. No
3. I don’t know
5. If so, how often do muscle cramps occur during dialysis session:
a. During every dialysis
b. Twice a week
c. Once a week
d. Once a month
e. Less often than once a month?
6. How long do muscle cramps last during dialysis session?
a. Up to 10 minutes
b. Up to 30 minutes
c. About 1 hour
d. Longer than 1 hour
7. Do you experience muscle cramps after dialysis:
1. Yes
2. No
3. I don’t know
8. If so, how often do muscle cramps occur after dialysis:
a. Every dialysis
b. Twice a week
c. Once a week
d. Once a month
e. Less than once a month?
9. How long do muscle cramps last after dialysis?
a. Up to 10 minutes
b. Up to 30 minutes
c. About 1 hour
Longer than 1 hour

## Data Availability

The original contributions presented in this study are included in the article material. Further inquiries can be directed to the corresponding author.
